# Overview of online pharmacy regulations in Saudi Arabia and the Gulf cooperation council countries and their impact on online pharmacy service providers in Saudi Arabia: a qualitative analysis

**DOI:** 10.3389/fphar.2024.1380231

**Published:** 2024-07-31

**Authors:** Basmah H. Alfageh, Norah O. Abanmy, Basma Y. Kentab, Omar A. Almohammed

**Affiliations:** ^1^ Department of Clinical Pharmacy, College of Pharmacy, King Saud University, Riyadh, Saudi Arabia; ^2^ Pharmacoeconomics Research Unit, College of Pharmacy, King Saud University, Riyadh, Saudi Arabia

**Keywords:** online pharmacy, E-pharmacy, GCC, Saudi Arabia, rules and regulations

## Abstract

**Background:**

Laws and regulations are needed to regulate the growing online pharmacy (OP) services. The main objective of this work was to provide an overview of the laws and regulations for OP services in the Gulf Cooperation Council (GCC) countries. In addition, the perception of how these laws and regulations in Saudi Arabia (SA) affect the online ordering of medications and health-related products from national and international OPs was explored.

**Methods:**

A secondary data collection through emails and a qualitative descriptive analysis was used to gain insight into the OP regulations in the GCC countries. Then, a qualitative study was carried out with semi-structured interviews to investigate the impact of these regulations on the practice and the market from the OP service providers’ perspective. The interviews were carried out with a sample of major OP service providers in SA, to represent the GCC countries. During the interviews, multiple open-ended questions were used to explore opinions about the OP regulations and how these regulations affected the practice. The interviews were then transcribed and thematically analysed.

**Results:**

Responses were mainly received from regulators in SA, Bahrain, Oman and United Arab Emirates (UAE). SA and UAE allow for offering of OP services as add-on service for existing community pharmacy, while UAE also allows for standalone OP providers. SA, Bahrain, and Oman allow online ordering of both over-the-counter (OTC) and prescription-only medications (POM) from international OP; a prescription is required for POM and quantities allowed should be no more than 3 months’ supply in case of SA and Oman while this was not specified in case of Bahrain. Invoice of purchase was also required for any POM to be released from customs in SA and Bahrain but not in Oman and UAE. Controlled medications were prohibited to be ordered online in SA, UAE, and Bahrain while it was allowed in Oman if the prescription was issued within 6-month, and the quantity dispensed was for 1 month only. Apart from online ordering of medications in these countries, no specific regulations existed to regulate ordering of other health-related products from local or international OPs. Whether Kwait and Qatar have regulations for OP could not be established due to lack of response. Two of the four interviewed representatives of OP service providers in SA were not aware of the existence of specific regulations for OP services. The representatives who were aware of these regulations were satisfied with them and found them beneficial for their business and for the patients at the same time. However, representatives raised concerns regarding the enforcement of regulations on international OP providers.

**Conclusion:**

The existing regulations for online ordering of medications are somewhat comparable between the GCC countries, with no specific regulations for ordering of other health-related products from local or international OPs. In SA, there is limited awareness of the existing regulations for OP services by providers. Nevertheless, the need for detailed regulations on certain aspects of OP services was highlighted, such as regulations for international OPs and importing medications for personal use.

## 1 Introduction

In developing countries, the law and regulations for online pharmacy (OP) services are still under development and the popularity of OP is much less than that in the United States, United Kingdom (United Kingdom), Europe or Canada. The development of laws that regulate the process of ordering prescription and non-prescription medications as well as herbal and other medicinal products has an important impact on public health (Fittler et al., 2013; Long et al., 2022). OP websites and businesses regulations are important to ensure patient safety and to provide a fair market among local and international pharmacies. Regulatory authorities in some countries have published policies to regulate the execution of online orders that include medications or similar products (U.S. FDA, 2022; National Association of Pharmacy Regulatory Authorities, 2024; General Pharmaceutical Council, 2024; European Medicines Agency, 2024a; Therapeutic Goods Administration, 2024). Such regulations are very crucial to ensure public safety and protect patients’ and providers’ rights. Having a well-founded regulation will protect against the distribution of counterfeit medications, sale of medications without legal prescription or sale of health-related products with misleading medical claims. Moreover, firm regulations will ensure consumers’ rights and prevent any fraud transaction or inappropriate data use by unlicensed local or international online stores. Pharmacists and other workers in this field should have an overall understanding of existing regulations to be able to execute online orders safely and effectively.

Many developed countries such as the United States, United Kingdom, Canada, some European countries and Australia have introduced different regulations to prevent counterfeit medicines from entering the legal supply chain system and directly reaching consumers (U.S. FDA, 2022; National Association of Pharmacy Regulatory Authorities, 2024; General Pharmaceutical Council, 2024; European Medicines Agency, 2024a; Therapeutic Goods Administration, 2024). These regulations include: introducing a common logo for legitimate OP providers and the need to display the OP’s registration number, the name of the owner and the superintendent pharmacist, as well as the physical address with relevant phone number(s) and email address. Furthermore, a valid and signed prescription from a healthcare provider is an essential element in this process, mainly for prescription-only medications (POM). Although these regulations have proven effective, there is a need to extend such regulations worldwide to overcome any counterfeit medication or rogue websites globally.

In the Gulf Cooperation Council (GCC) countries, which include Saudi Arabia (SA), United Arab Emirates (UAE), Bahrain, Kuwait, Oman, and Qatar, the popularity of OP services gained little attention as the number of published papers on this topic is limited. Those published in SA or UAE focused on the existence of such services, consumer experience with OP services, or the safety issues related to these OP purchases (Alfahad et al., 2015; Abanmy, 2017; Ashames et al., 2019; Alwhaibi et al., 2021; Jairoun et al., 2021; Almohammed et al., 2023; Almeman, 2024). The extent of use of OP services in SA has increased from around 3% in 2017 to 60% in 2023 (Abanmy, 2017; Almohammed et al., 2023), while the figure is much lower in UAE as the extent of use of OP was 10% in 2019 and increased to 31% after the COVID-19 pandemic (Ashames et al., 2019; Jairoun et al., 2021). Data on the use of OP services in other GCC countries are lacking and there is a need for further research to build-up the evidence about the topic. No previous studies were conducted to assess the laws or regulations for the OP services in the GCC countries.

Having clear and specific regulations for OP services in the GCC countries will regulate this market from the business perspective. Moreover, the regulatory authorities need to disseminate these regulations to the public to inform them of their rights and how they can safely benefit from these OP services. The existence and dissemination of such regulations will enhance the patients and consumers’ awareness of their rights and enable them to assess the legitimacy of OP websites and purchase medications online with confidence. Moreover, the understanding of the concerns and challenges about this market is needed in order to improve the current status while protecting both patients’ rights and pharmacists’ jobs in this field. This may also help policymakers set guidelines to manage the OP business in this region of the world in order to protect local citizens and residents in the GCC countries as well as to protect local and regional business from losing its share in the market which means losing local jobs and investments in this field for international businesses.

This study aimed to provide an insight into the regulations governing OP services in the GCC countries. Collecting and comparing the regulations for OP services among the GCC countries and between GCC countries and other countries worldwide was carried out in order to evaluate the current status and identify areas for improvement in regulations. We also aimed to explore the views and concerns of a sample of OP service providers in SA in relation to OP regulations to help identify challenges and opportunities in this area.

## 2 Methodology

### 2.1 Study design and setting

This was a qualitative study that was conducted in SA with a local and regional perspective that included SA, Bahrain, Kuwait, Oman, Qatar and UAE. With little known about what regulations govern OP service provision in the GCC countries and how they compare to international regulations, as well as a lack of data on service providers’ views and the challenges they encounter, qualitative methodology was considered the most suitable approach to achieve the study objectives. The study involved a secondary data collection with a qualitative descriptive analysis of existing OP regulation documents in the GCC countries, to gain insight about the OP regulations in these countries. In addition, semi-structured interviews were conducted to investigate the impact of these regulations on the practice and the market from the OP service providers’ perspective.

### 2.2 Target sample

The regulatory authorities for online or other forms of medication distribution in the GCC countries were the targeted sample for the regulations’ comparison part of the study. Interviews were carried out with a sample of major OP service providers in SA, to represent the OP service providers in the GCC countries. Major OP service providers represent pharmacy chains that operate a large number of community pharmacies in multiple cities across SA and provide OP services. To the best of our knowledge there is no publicly accessible official list of OP service providers in SA. Based on the researchers’ familiarity with the local Saudi context and utilizing a purposeful sampling approach, the largest six major OP service providers were identified and invited to participate in the study. SA is the largest among the GCC countries, in terms of population, area, and economy and including OP providers from SA would represent the majority of the OP providers in the GCC countries.

### 2.3 Data collection, data source, and study variables

Regulatory documents were obtained from the relevant regulatory authorities in the GCC countries through official contact via the Gulf Health Council (GHC) or through direct correspondence with researchers in these countries. The GHC was approached by email and in-person with a request to contact the relevant drug or pharmacy related authorities in all GCC countries to obtain information regarding the OP regulations. Two reminder-emails were sent to the GHC to elicit the response of those who did not respond to the first GHC email. Some GCC countries, namely, Kuwait, Qatar and UAE, did not respond to the GHC email or did not provide any documents. In order to ascertain whether regulations existed for OP, we initiated direct correspondence with researchers from these GCC nations to ensure the exhaustiveness of our inquiry. This approach identified the existence of publicly available regulations in the UAE that were not delivered to us via the GHC. To conduct a thorough examination of the regulations, we reviewed the regulations that have been published online in countries with well-established legislation for OP, specifically the United States, United Kingdom, Canada, Europe, and Australia. This enabled a more comprehensive and accurate evaluation and comparison of the existing standards for OP practices in the GCC countries.

Contact persons at major OP service providers in SA were approached and an explanation of the study purpose and procedures was provided. Representatives identified by OP service providers were interviewed to obtain their insight on the current regulations for OP services. A semi-structured interview guide was used to understand the participants’ concerns and perspectives towards the available regulations and to explore the challenges affecting their practice with regards to these regulations. The interview guide was developed based on the authors’ prior experience in qualitative methods and online pharmacy services’ research. It was then piloted with one representative of OP providers. This interview was included in the final analysis as no change was made to the interview guide after piloting. The interviewees were asked about the regulation for OP services in SA only and no questions were asked about the regulation in the rest of the GCC countries. All interviews were conducted online via Zoom^®^ platform. We planned to continue interviewing until data saturation was achieved.

### 2.4 Ethical considerations

The ethical approval for this study was obtained from the standing committee for Scientific Research Ethics in King Saud University (Reference no. KSU-HE-23-788). Representatives of the OP services providers were asked to complete an online consent form prior to the interview to ensure their employers’ approval for their participation in the study. Participants were informed that the interview will be audio recorded and that data will be used for this research only.

### 2.5 Data analysis

The regulations in the GCC countries were summarized and described based on their availability to allow for comparison among the countries. The interview recordings were anonymised by assigning codes to differentiate between participants, e.g., PR1 (Pharmacy Representative 1). All interviews were transcribed verbatim, and then subjected to thematic analysis by identifying common themes and including relevant illustrative quotes (Clarke et al., 2016). One interview was double-coded independently by two authors who then met, discussed, and agreed on the main themes as well as subthemes. Analysis was then performed on the remaining interviews using the agreed-upon themes.

## 3 Results

### 3.1 Regulations in the GCC countries

The regulatory authorities in SA, Bahrain, Oman, and the UAE responded to inquiries about the regulations governing OP services in their respective countries, while no responses were received from Kuwait and Qatar. Direct correspondence with researchers identified further regulatory documents from the UAE. OP regulations were either listed in a specific regulation document for OP services or listed as part of single or multiple larger regulatory documents for providing health services in the relevant country. After reviewing the data received from the regulatory authorities or researchers in the GCC countries, it was apparent that generally no specific regulations for OP exist that are comparable to the regulations in developed countries, which will be discussed later on. Overall, the available regulatory documents were more like policies and procedures or technical documents that focus on the regulations for the process of ordering from OPs.

#### 3.1.1 The regulations in SA

In SA there are no OP that provide their services only online. However, there are some community pharmacies that accept online orders for medications and other nonpharmaceutical products through their websites. The regulations of online orders are the same as those for the usual dispensing of medications from community pharmacies or brick-and-mortar pharmacies. Ordering medications from international OPs is allowed for both over the counter medications (OTC) and prescription-only medications (POM) after obtaining the permission of the Saudi Food and Drug Authority (SFDA). The main requirements that allow the release of POM from Saudi Customs are to provide a detailed prescription that includes patient’s name, physician’s name, as well as drug name, dose, route of administration and frequency in addition to the invoice for buying the medication. The quantity of the POM that is allowed to be ordered and dispensed through OP is only for up to 3 months’ supply. Although the POM is allowed to be ordered from online pharmacies, the ordering of controlled medications is totally prohibited. In case of OTC medications, health or beauty products, and supplements, it is required to fill the personal clearance request form, provide the invoice for the ordered products, bill of lading and a proof of personal identity (Saudi FDA, 2024).

#### 3.1.2 The regulations in Bahrain, Oman, and UAE

The requirements to release any medicinal products from the Bahraini customs if ordered from OP outside Bahrain were the same as in SA, except that there was no mention of any duration of treatment or quantity. However, commercial quantities are prohibited and these online orders should be for personal use only. The regulations in Oman were similar to SA as it allows for online ordering of POM with a valid medical report and the OP may dispense up to 3 months’ supply. However, controlled medications can be dispensed in Oman through OP with a valid prescription that was issued no more than 6 months ago, and the quantity can be enough for up to 1 month only. Besides that, no information was received in regard to local practices of OP from Bahrain or Oman. UAE allows for OP to practice locally as a standalone pharmacy or add-on service to the usual community pharmacy whereas the regulations are similar to the usual dispensing of medications from community pharmacies. However, regulations for medication ordering from international OP was lacking in UAE. (Table 1). Ordering other health and beauty products and supplements was not part of the regulations in Bahrain and Oman, while the regulation in the UAE allowed for the online ordering and delivery of these products.

**TABLE 1:** Online pharmacy regulations in SA, Bahrain, UAE, and Oman.

	SA	Bahrain	UAE	Oman
Regulatory authority (source of regulations)	Saudi Food and Drug Authority (SFDA)	National Health Regulatory Authority	Dubai Health Authority	Directorate General of Pharmaceutical Affairs and Drug Control
POM^a^	Yes	Yes	Yes	Yes
OTC	Yes	Yes	Yes	Yes
Quantity specified for international orders	For 3 months’ supply	No specific quantity, but not for commercial use	No specific quantity, but not for commercial use	For 3 months’ supply
Invoice for international OP orders	Yes	Yes	No specific regulation for international ordering of medications	No
Controlled medications	No	No	No	Yes, with valid prescription (issued within 6 months), and quantity for 1 month

^a^ Ordering POM, is allowed providing that patient has a valid prescription.

SA, saudi arabia; UAE, united arab emirates; POM, prescription-only medications; OTC, over the counter medications.

### 3.2 Providers’ perception of regulations of online pharmacy services in SA

A total of six major OP providers in SA were invited through emails or phone calls, of whom four responded and participated in this study. These four OP providers launched their OP services relatively recently with durations spanning from one to 6 years. In addition to cosmetics, health supplements, and OTC medications, two OP providers offered the delivery of POM while the remaining two offered it as a pickup service at the time of the interviews, with future plans for delivery. None of these providers offered the delivery of controlled medications. Virtual pharmacist consultation was offered for free by three out of the four OP providers who participated in the study, and the fourth was planning to offer it soon and in a comparable way. The characteristics for the pharmacy chains that provided OP services in SA and participated in the study were summarized in Table 2.

**TABLE 2:** The characteristics for pharmacy chains that provided OP services in SA.

OP representative	No. of branches across SA	Year of launching OP service	Products available for online purchase	Delivery of POM	Virtual pharmacist consultation
PR1	+200	2021	OTC medications, cosmetics, babies supply, vitamins and supplements and health-related products	Not available	Not available
PR2	1,200	2018	POM, OTC medications, babies supply, vitamins and supplements and health-related products	Available	Available
PR3	+150	2023	POM, OTC medications, cosmetics, babies supply, vitamins and supplements and health-related products	Not available	Available
PR4	910	2019	POM, OTC medications, babies supply, vitamins and supplements and health-related products	Available	Available

OP, online pharmacy; SA, saudi arabia; OTC, over the counter; POM, prescription-only medications. The source for this information were mainly the interviewees or the website for these chain pharmacies.

After analysis of the four interviews’ transcripts, four major themes were revealed. These themes include: regulations that govern online service provision, opinion on existing regulations, challenges to OP service providers, and suggestions to improve the regulations of OP services. A detailed description of these themes with supporting quotes are presented below.

#### 3.2.1 Regulations that govern online service provision

##### 3.2.1.1 Awareness of specific guidance for online pharmacy services

Two of the four interviewed pharmacy representatives were not aware of the existence of specific regulations for OP service provision (PR1 and PR3). Their companies were extending the Ministry of Health (MOH) Regulations of Pharmaceutical Products and Facilities Law (Ministry of Health, 2024), ​ e.g., the requirement of a valid prescription for prescription medications, to design and provide their OP services. The other two OP representatives (PR2 and PR4) mentioned the specific MOH regulations and elaborated on a number of items within these regulations.

PR3: *“As for online laws, or online pharmacies, until now, clear laws have not appeared that clarify the work, and no clear policies for dealing in online pharmacy have emerged.”*

PR4: *“The most prominent regulations for virtual pharmacies. You must have a website. It must have license numbers, for example, the commercial registry number, the SFDA license number, and the MOH license number must all be presented on the website. There are also some standards, such as: the products, their pictures on the website and the application, and having an electronic website or application to display and sell the products.”*

PR1: *“We set a frame for ourselves. Anything we cannot prescribe offline, we cannot prescribe online. For example, antibiotics are forbidden to be taken without a prescription.”*

It has been acknowledged that multiple governmental authorities have rules in the legislation of online pharmacies in SA. The SFDA, MOH, Ministry of Commerce and Customs Authority were all mentioned by some of the pharmacy representatives.

PR2: *“There are more than one regulating body, and maybe three. Certain medical services, such as prescribing through electronic devices such as robots, the dispensing machines found in airports and streets, the multi-dose packaging systems and the service for refilling dispensed medications, these of course have laws.”*

PR3: *“The one who can decide on this matter is neither the MOH nor the Ministry of Commerce. The one who can decide on this matter is the Customs Authority.*

PR1: *“For example: if you are importing a prescribed medication for personal use, e.g., two packs, it will pass through the customs for declaration. The customs declaration will ask you for a prescription. On other hand, narcotic medication no way that can enter SA even if it has a prescription. For this reason, even customs have a list of certain items. But is there a written and organized framework and do we have an alignment between the Customs Authority, the MOH, and the SFDA? This is what we need to organize.”*

##### 3.2.1.2 Adherence to other governmental regulations and requirements

Although two of the four interviewed OP representatives were not aware of the specific regulations for OP services, all participants confirmed that uploading a valid prescription is required to provide prescription medications through the OP services. Three of the interviewed OP representatives were actively dispensing prescription medications at the time of the study and the fourth was planning to begin the service in the near future. It was clear that they were applying the existing regulations for physical pharmacies (brick-and-mortar) when dispensing POMs.

PR2: *“I mean, if you are going to buy antibiotics from the pharmacy, you will need to upload a prescription, meaning you will need to submit a prescription, which means the same rules. Almost the same products found in the pharmacy are available online, and it imposes the same rules and regulations that govern medication dispensing in the physical store, also present in the online store.”*

PR3: *“An online pharmacy, just like a physical store. Behind the screen there is a pharmacist, and there are assistants with him … Okay. It is definitely obligatory, that he dispenses medicines and the needs based on the prescription that comes. Exactly like physical pharmacy rules.”*

PR4: *“In addition, in order for us to dispense any prescription to the customer, we must have the prescription, it must meet the conditions requested by the MOH.”*

Governmental inspection on the uploaded prescriptions was also discussed with the participants. It was clear that adherence to the regulations regarding prescriptions uploading depended on the providers’ policies and the extent of their commitment.

PR3: *“It depends on the company itself. The extent of the company’s commitment to the policies and laws of the MOH. There are people who commit and are very careful because in the end this is a responsibility.”*

##### 3.2.1.3 Availability of internal policies

Extra internal policies were mentioned by some of the interviewed OP representatives. Some required a direct contact between a pharmacist and the patient, including asking them several questions to confirm that the ordered medication is suitable for their case and would not lead to any drug-drug interaction. Also, obtaining the original prescription from the patient upon the delivery of the order was one of the extra internal policies that were discussed.

PR2: *“In our institution, certain questions must be asked by an expert pharmacist consultant to the patients before providing these medications. We have a rare thing called the ‘WWHAM’ in which the pharmacist must ask the patient who is this medicine for, why, how old is the patient? What are the other medications he/she takes? To avoid drug-drug interaction, and this is a special thing between us*”.

PR3: *“Here, when we came to make the strategy or the policy for the online store, we required the communication with the customer. This means that there is a stage in which there is a direct phone call with the customer. The customer can request the order and we will get the order and prepare it. Before the delivery service, we communicate with the customer directly. The pharmacist communicates with the customer, verifying the information that he is really the customer who ordered this product, okay, and he also tries to engage in any kind of dialogue with him so that he ensures that the customer does not have any contraindication.”*

#### 3.2.2 Opinion on existing regulations

##### 3.2.2.1 Clarity and completeness of regulations

The two OP representatives who were aware of the existence of specific OP regulations agreed on the clarity and comprehensiveness of the policies. In contrast, the remaining two OP representatives asked for specific OP regulations as they were not aware of its existence, they also highlighted the need for regulations that applied to international competitors and drug stores, policies for providing narcotics and control dugs online and policies for importing medications for personal use versus commercial use.

PR4: “*All the regulations are comprehensive and meet the requirements*.”

PR2: *“From my point of view, things are currently very clear and the licenses are very clear. Process is clear in dealing with complaints and following up on complaints, which means attention from government agencies*”.

PR1: *“According to the law, pharmacies licensed to dispense narcotic must have a Saudi pharmacist available. The original prescription must be provided. Okay, we do not know how to apply the rules online. So, we made a list and hid the categories that were originally narcotic from the website. But if there is a frame or a hint, the government says there are requirements from one to ten in order to dispense it online we will adhere to it. But currently, I am afraid that if I work on personal effort, someone will come and file a lawsuit, then we lose the opportunity to provide the service to the customer.”*

PR1: *“This needs to be linked between customs, the MOH, and SFDA. In customs clearance, any item that is more than two or three pills must pass the SFDA and its source must be certified and not for commercial use. You protect the internal market .”*

PR3: “. *Many problems have arisen with the use of products from international online stores. Because there is no control over them inside SA. The one who can decide on this matter is neither the MOH nor the Ministry of Commerce. This matter is decided by the Customs Authority.”*

##### 3.2.2.2 Effect of regulations

The OP representatives agreed on the benefit of having OP regulations. They mentioned that they had positive impact on their business and were helpful for the patients as well.

PR4: *“I see that the presence of these regulations makes the service easier for us and makes everything clear.”*

PR2: *“I do not see any negative impact of it. On the contrary, the clearer and organized regulations there are for each place, the more beneficial it is for the institution and the provider, and also for the patients.”*

#### 3.2.3 Challenges to online pharmacy service providers

OP representatives discussed a number of regulatory challenges associated with the provision of OP services. The challenges can be broadly classified into three categories: the lack of enforcement of regulations on international competitors and discount stores (specialty retailers that primarily focus on the sale of cosmetics and skincare/haircare items), the regulations related to the provision of medical information and consultations, and the provision of narcotic or controlled medications through online services.

##### 3.2.3.1 Lack of enforcement of regulations on international competitors and discount stores

OP representatives attributed the ability of international online providers and local discount stores to offer unparalleled discounts to the fact that regulations governing local community pharmacy businesses did not apply to these entities. This understandably affected their sales and profit. One pharmacy representative mentioned the fact that online sale of medications represented a small percentage of the total online sales, which might explain why participants considered international online providers and local discount stores major competitors.

PR1: *“The importation of items from abroad that are not certified by the SFDA, this affects you. The second thing is, the importation of cosmetic items from X, Y, and Z, not the main source licensed by the SFDA. So, they are sold with 70% and 80% discount. We cannot compete with these people because we bring [items] from the main source and the certified source so the price is forced on us. […] this threatens the pharmacy sector in the Kingdom.”*

PR3: *“Lately, somethings appeared which they call discount shop, stores, which are these who offer unimaginable discounts. Okay? These do not have regulations that govern them to this day. […] they come and offer unimaginable discounts, so these have a negative effect on us.”*

PR3: *“The percent of medicine sales in the online store, especially in the Saudi pharmaceutical market, is not large, not very much…in comparison to the non-pharma [products].”*

##### 3.2.3.2 Regulations related to the provision of medical information and consultations

One OP representative discussed how his company was planning to provide medication information to patients online and to offer the opportunity to consult with a physician or a pharmacist but was concerned about how the regulations govern the provision of such services.

PR1: *“Is there an official link from the Saudi MOH that has the interactions of drugs? So that it could give me…if you add to the basket this item with that item, it would give you an alarm or a notification that tells you* “*No, this drug is contraindicated with this drug,*”*. automatically, with no outside interference. I honestly do not know if this is available in SA or not.”*

PR1:*“This will be in the second phase. There will be consultation not only with a pharmacist, but with a physician. But to reach this phase, we need to look into the legality of it.”*

##### 3.2.3.3 Provision of narcotic or controlled medications through online services

The provision of narcotic or controlled medications through online services was also identified as a challenge by pharmacy representatives as the regulations that govern this were not clear.

PR1: *“I have a concern. We need to look at the governmental regulations. How do we ensure…, of course until now we have not gotten into narcotics, that the prescription will be dispensed once? […] So, we have closed this matter because we do not know how to ensure its regulatory [status].”*

#### 3.2.4 Suggestions to improve the regulations for online pharmacy services

The OP representatives offered a number of suggestions that they believed could improve OP regulations in SA. These included having specific regulations that are continuously reviewed and updated, having consistent regulations that apply to all online retailers to prevent malpractice, and requirement for prescriptions to be dispensed through an MOH portal.

PR1: *“*There *should be clear executive regulations for the online [services] that we can adhere to. That whoever does business or opens an electronic online store should be involved in the field on the ground. They should have a pharmacy on the ground. The owner of the site must be a S. pharmacist, because* the *law says this same thing for the [community] pharmacy.”*

PR2: “…there is continuous spread of the idea of online [services] much more than the physical or the offline [services]. So, this requires a review of the progress that is happening during this period […]. So, there should be agility and review of these regulations in line with the development that could happen…”

PR2: *“…*There *should be specific guidelines, not anyone with a pharmacy can open an online store. There should be specific guidelines that all people follow to the best of the customer’s interest and to prevent* malpractice *in such thing.”*

PR4: *“If* they *[discount stores] were to comply with the MOH and the MOH regulations, they would certainly be very careful about not ruining the market.”*

## 4 Discussion

From the first glance at the available regulations for OP services in the GCC countries, we can observe that there are no dedicated entities in the regulatory authorities that regulate OP operations. In case of online orders for medications from websites outside the GCC countries, the custom services are the only entity that control the release of the ordered medications based on the regulations published by drug authorities in the GCC countries. There are some regulations that are applied by the Saudi customs when processing orders that involve importation of medication internationally when it reaches the borders which includes, but not limited to, providing a valid prescription for POM and the quantity being suitable for personal use only.

In SA as well as the other GCC countries, there are no business institutions that provide pharmacy services through OP channels only. The existing laws and regulations in SA obligate providers to have a licensed physical pharmacy to obtain the license for providing services online through a website or smartphone application that enable it to receives and process online orders. Although this is applicable for local OP, it is not applied for international OPs that can deliver medications and other health-related products without the need for a physical pharmacy in SA. We believe this variation in regulation between the local and international OPs is an additional hurdle for new local OP and a threat for existing local OPs.

The regulations on type of medications dispensed through licensed OP were generally similar all over the world including the GCC countries, which allow the dispensing of POM if a valid prescription is provided and ban the dispensing of narcotic medications. However, regulations for online medication ordering are more comprehensive and well established in developed countries such as United States, United Kingdom, Europe and Canada than in the GCC countries. This could be due to the fact that the OP services have been offered in these countries since approximately two decades while it is still growing in the GCC countries (Long et al., 2022). In some low-income countries such as Kenya, Nigeria and India, no specific framework for OP has been legislated yet (Miller et al., 2021).

In contrast, looking deeply into regulations from countries with more mature regulations for OP we find that multiple measures have been taken to ensure safe online purchase of medications in the United States, United Kingdom, Europe, Canada and Australia (U.S. FDA, 2022; National Association of Pharmacy Regulatory Authorities, 2024; General Pharmaceutical Council, 2024; European Medicines Agency, 2024a; Therapeutic Goods Administration, 2024). One of these measures was to ensure that customers have a method for identifying whether the OP is legitimate and registered. In the United States, the USFDA indicated that checking OP’s license should be through the state board of pharmacy website (U.S. FDA, 2024), while in Canada, United Kingdom and Europe through displaying a specific obligatory logo on the OP website (European Medicines Agency, 2024a; General Pharmaceutical Council, 2024; National Association of Pharmacy Regulatory Authorities, 2024). In addition, the Canadian International Pharmacy Association (CIPA) provides the accreditation for legal OP websites and each CIPA member prominently displays the official CIPA red oval certification mark on their website (Canadian International Pharmacy Association CIPA, 2024). While the National Association of Boards of Pharmacy (NABP) is responsible for granting an internet domain to legal online pharmacy (.pharmacy) as tools to identify legitimate OP in Canada (National Association of Boards of Pharmacy, 2024). In Australia a trusted OP will have a web address that ends in*. com.au*, ask the customer to send a prescription, give details of Quality Care Pharmacy Program accreditation or registration with a state pharmacy council and lastly give a phone number for the customer to talk to a pharmacists (Therapeutic Goods Administration, 2024). To ensure that medicines are safe, a unique identifier and anti-tampering device is placed on the packaging of most medicines for human use in European countries (Naughton et al., 2015; European Medicines Agency, 2024b). Regarding the purchase of drugs from OP, as previously indicated most regulations in these countries permit the sale of POM online while prohibiting the sale of narcotics and controlled substances (U.S. FDA, 2022; National Association of Pharmacy Regulatory Authorities, 2024; General Pharmaceutical Council, 2024; European Medicines Agency, 2024a; Therapeutic Goods Administration, 2024). Moreover, the public in these countries are educated through official websites for the regulatory entities in each country about additional criteria to help customers differentiate between legitimate and non-legitimate OP, such as: OP asking for doctor’s prescription, having a local physical address and telephone number, and having licensed pharmacists to answer customers’ questions. That being said, none of these requirements exist in the GCC countries and having such regulations may help organize this fast growing market in the future.

In the GCC countries, no strict regulations were applied to the ordering of medications and other beauty and health supplements from online resources other than the local licensed OP stores, such as international OPs and online stores. This deficiency in regulations may lead to public health concerns, such as the sale of POM without a prescription, or the sale of substandard medications and medicines or health-related products with misleading medical claims (O Hagan and Garlington, 2018). Other governmental and public concerns that may arise and threaten the consumers are the occurrence of fraud or inadequate data protection by the online stores (Almohammed et al., 2023). These are major concerns that need to be taken into consideration when formulating local regulations for OP practices in the GCC countries specifically and other countries as well.

Applying firm regulations on ordering medication and other health-related products from any source will ensure importing these products safely to patients and consumers and protect the local market from collapsing due to the unjust competition between the local OP providers and the international retailers. A recent research showed that pharmacists in Ghana shared similar opinions on the challenges that affect providing OP services as the local OP representatives in our study (Eab-Aggrey and Khan, 2024). Lack of effective regulations and the unintended consequences were of the major concerns reported by the study participants.

The United Nations Sustainable Developed Goals (UN-SDG) report has indicated that the GCC countries have some challenges regarding Goal no. 3, which is related to good health and wellbeing (Umar and Umeokafor, 2022). We think that by offering more OP services as well as using the technology to provide tele-health/tele-medicine services, the GCC countries may have an opportunity to improve the health of their citizens and residents and ensure providing better healthcare services which will have a positive impact on achieving higher score on this goal. Currently, there are challenges in the region with Goal no. 3, the score of the goal in different GCC countries is increasing but it is increasing in a sufficient pace to achieve the goal by 2030. However, the introduction of regulation for online health services, including OP services, and establishing these services locally would facilitate the reach of these countries to their goals by 2030.

To our knowledge, this study is among the first studies that shed light on the current rules and regulations for OPs in the GCC countries. The qualitative part employed in this study explored the perception of OP service providers in SA about these rules and regulations and highlighted concerns about providing OP services in SA. We believe that the findings of this study may help in evaluating the current status and pave the road to improve the current situation for OP services in the region and draw the attention of the legislators to the challenges that face the local market.

Some limitations and many challenges were encountered in this study. A major challenge pertained to the ease of access to regulatory documents which are not always publicly available. We opted for official contact with the GCC countries through the GHC in order to ensure the completeness and accuracy of data, but encountered lack of response or incomplete documents from some countries. However, this challenge and limitation was partially overcome by personal correspondence with researchers in these countries. In addition, comparing the GCC regulations with well-established legislation for OP from developed countries has strengthened our findings by highlighting the deficiencies that require further improvement. Finding the pertinent OP regulations was more complicated in a country like the UAE that have both state and federal regulations. Most of what we received and included in this manuscript about the UAE were pertaining to the states of Dubai or Abu Dhabi, so the regulations in regards to the UAE should be interpreted considering this limitation. Furthermore, the involvement of multiple authorities in the regulation of OP services, either local or international, result in fragmented regulations that become difficult to compile and analyze. Because of the time constraints for the project and the low response rate from the OP providers, as only four representatives from OP service providers in SA responded and agreed to participate in this study, we were unable to conduct additional interviews and we cannot guarantee that data saturation was achieved. However, the four participants were from major OP service providers in SA with years of experience in the field. Moreover, the validity and reliability of the findings from interviews were enhanced by the independent double-coding of data, keeping an audit trail of all data generated throughout the study (e.g., audio recordings, transcripts, analysis), and recording notes on thoughts and impressions during and after each interview to maintain reflexivity. Lastly, the extent to which the findings relating to the perspectives of Saudi OP providers can be applied to other GCC countries may be limited by variations in the local context.

That being said, researchers interested in pharmacy or health regulatory research, especially when it involves multiple countries, should be aware of the need to utilize multiple resources to ensure complete data collection. Researchers should consider the time frame required to conduct such research considering the time needed to gather responses, send reminders if necessary, and interview participants as needed. Future research may focus on exploring the opinions of OP service providers across the GCC utilizing a quantitative approach. The views about international OP service providers and its impact on local OP service providers also warrant further exploration as no literature exist about this matter. Research is also needed on how existing regulations may be updated, expanded, unified among states or countries, and disseminated which will require engagement and open communication with key stakeholders and regulatory authorities both locally and internationally.

## 5 Conclusion

Although OP services provide an opportunity for consumers over the traditional pharmacy, firm regulation of these services is vital to ensure consumers’ safety in the first place. This study revealed that regulating online ordering of pharmaceutical products from local licensed OP websites alone is not enough. There are many other local and international online resources providing health-related supplements and beauty products and sometimes even POM which need to be also regulated. Applying unified regulations in addition to frequent monitoring of the licensure system would reduce the number of illegitimate OP providers and protect both the consumer and local market.

## Data Availability

The raw data supporting the conclusions of this article will be made available by the authors, without undue reservation.
